# 771. COVID-19 on the Line: A Significant Increase in CLABSI in Hospitalized Patients with COVID-19 at a Major Teaching Hospital

**DOI:** 10.1093/ofid/ofab466.968

**Published:** 2021-12-04

**Authors:** Pishoy Haroun, Michael Ben-Aderet, Meghan Madhusudhan, Matthew J Almario, Ryan C Raypon, Sharon E Fawcett, Jonathan Grein

**Affiliations:** 1 UCLA, Sherman Oaks, CA; 2 Cedars Sinai Medical Center, Los Angeles, CA; 3 Cedars-Sinai Medical Center, Los Angeles, CA

## Abstract

**Background:**

We observed an increase in central line associated bloodstream infections (CLABSI) associated with the 2020 COVID-19 pandemic and performed a retrospective analysis to better understand the impact of COVID-19 on CLABSI rates.

Figure 1. CLABSI rate in 2019 vs CLABSI rate in 2020

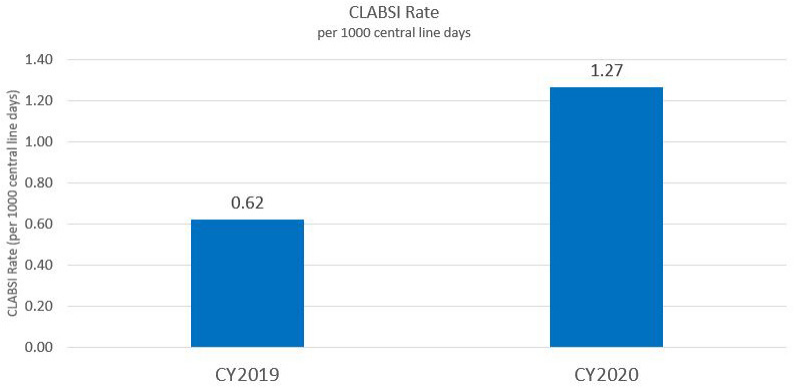

A comparison of CLABSI rates (displayed in infections/1000 catheter days) in all adult inpatients at our institution for calendar-years 2019 and 2020

**Methods:**

Retrospective review was done for all CLABSI in adults meeting National Healthcare Safety Network (NHSN) criteria in 2020 at an 889-bed teaching hospital. CLABSIs in encounters with PCR-confirmed COVID-19 (COVID CLABSI) were compared with CLABSIs in encounters without a COVID diagnosis (non-COVID CLABSI). As a secondary analysis, we also reviewed all CLABSI occurrence in 2019. Characteristics were compared using Mid-P Exact (Poisson) and Chi Squared (categorical) Tests. Subjective data collected by infection preventionists during real-time case reviews with clinical staff of each CLABSI was also reviewed.

**Results:**

In 2020, the rate of COVID CLABSI (CLABSI/1000 catheter days) was 6.6 times greater than the rate of non-COVID CLABSI (5.47 vs. 0.83, p< 0.001). In the COVID CLABSI group we observed higher rates of occurrence in the ICU setting (94% vs 28%, p< 0.001), in house mortality (53% vs 26% P=0.0187), presence of arterial lines (91% vs 20%, p< 0.001) and increased number of catheter lumens (4 vs 3, p< 0.001). No significant difference was observed in the distribution of pathogens. No significant differences were observed between 2019 CLABSI and 2020 non-COVID CLABSI. Real-time case reviews identified changes in nurse staffing, increased nurse: patient ratios, delays in routine central line dressing changes, and inconsistent use of alcohol-impregnated port protectors as possible contributing factors.

Table 1. 2020 COVID CLABSI vs 2020 non-COVID CLABSI

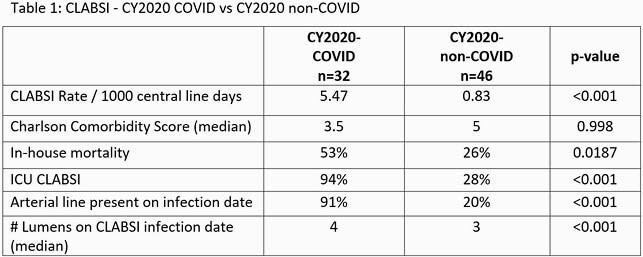

A comparison of selected patient and catheter characteristics in COVID CLABSI vs non-COVID CLABSI in 2020

Table 2. 2019 CLABSI vs 2020 non-COVID CLABSI

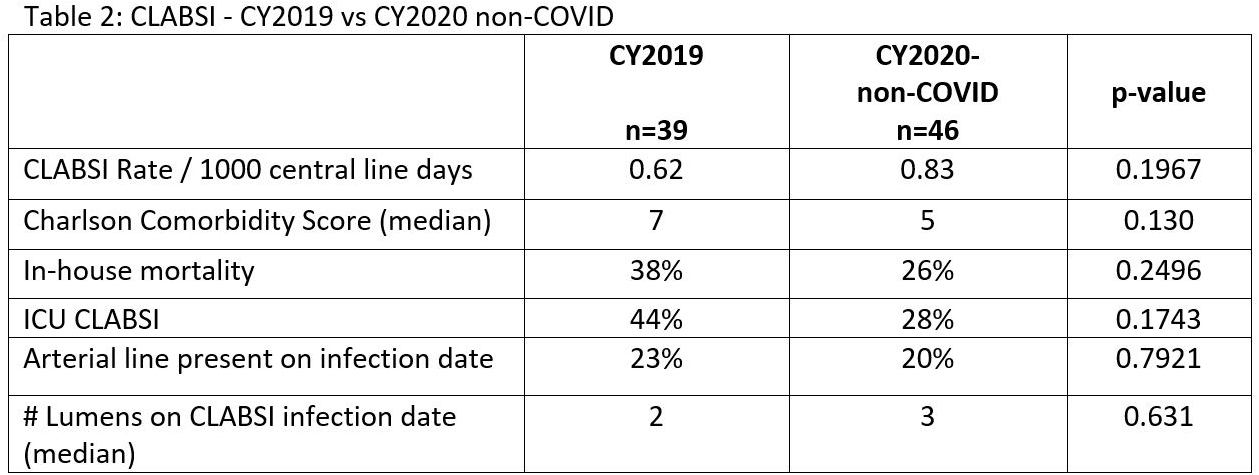

A comparison of selected patient and catheter characteristics in CLABSI in 2019 vs non-COVID CLABSI in 2020

Figure 2. CLABSI rate in 2019 vs COVID CLABSI and non-COVID CLABSI in 2020

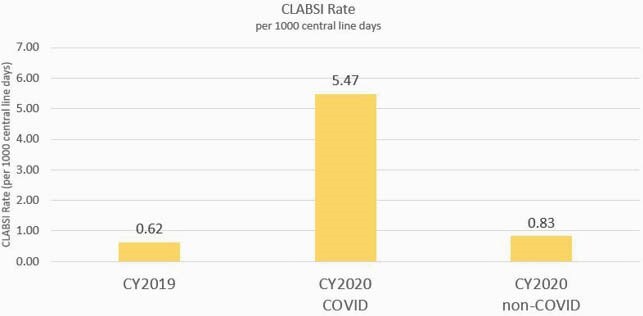

A comparison of CLABSI rates (displayed in infections/1000 catheter days) in all adult inpatients at our institution for calendar years 2019 and 2020, with the infections in 2020 divided into those that occurred during an encounter with a PCR -confirmed diagnosis of COVID-19 and those without.

**Conclusion:**

We observed a dramatically higher rate of CLABSI in patients with COVID-19 in 2020, while the rate of CLABSI in patients without COVID-19 remained unchanged from the year prior. Higher rates of ICU admission, critical illness, increased numbers of lumens, increased presence of arterial lines, nurse staffing changes, and gaps in routine line prevention processes associated with emergency measures in the COVID-19 cohort ICU may have contributed to this finding. Further work is needed to better understand how to minimize process-related disruptions in central line care during a hospital response to a pandemic.

**Disclosures:**

**Jonathan Grein, MD**, **Gilead** (Other Financial or Material Support, Speakers fees)

